# Supplementary material for: Determinants of Communication Failure in Intubated Critically Ill Patients: A Qualitative Phenomenological Study from the Perspective of Critical Care Nurses
Source: Healthcare (Basel). 2023 Sep 28;11(19):2645. doi: 10.3390/healthcare11192645 (PMC10572283; doi:10.3390/healthcare11192645)
Supplement: Supplementary file 1 [file healthcare-11-02645-s001.zip › supplementary file S1 revised.pdf]

Supplementary file S1. Semi-structured interview guide.

For the development of the interviews, a certain degree of structuring was chosen to ensure discursive content related to a set of central themes, which would later allow for cross-case comparison[(39)]. With this intention, we generate a guide of topics, based on the literature review[(38)] to propose to participants during the interview.

Two pilot interviews were conducted prior to the start of data collection, which were not included in the analysis. One of the objectives of the pilot interviews was for the principal investigator to become familiar with the interview script and focus, as propose by Kvale (cited by Bryman[39]). Additionally, the aim was to test the clarity of the questions and, if necessary, modify the initial script. Although the initial question script remained unchanged after the pilot interviews, it was further clarified as the interviews and initial analysis progressed. This iterative process resulted from the open and discursive nature of the interviews, as indicated by Bryman[(39)]. We added some questions and simplified others, resulting in a final script for both groups of informants, which is reflected in the following table.

The interview was initiated by a general question on the care of awake and intubated patients and carried out with an in-depth examination of the elements involved in the communication process with such patients. The interviewer used the script as a reminder of the subjects to address, never as a closed protocol as to contents and order.

1. You are an ICU professional and usually care for mechanically ventilated patients who are awake or on low sedation. Could you explain in general terms how you would describe your experience in care of those patients?
2. What kind of communication issues do you have with this kind of patients?
3. From your point of view, what factors impact on the communication impairment of those patients?
4. In general, do you think that nursing professionals foster communication with those patients? What kind of strategy do you personally use to communicate with them? Do you use it frequently? If you don't, why do you think you don't use it?
5. Imagine you had an ideal setting for clinical practice, what actions do you think could improve communication between the patient with communication impairment and the professionals?
