# Supplementary material for: Determinants of Communication Failure in Intubated Critically Ill Patients: A Qualitative Phenomenological Study from the Perspective of Critical Care Nurses
Source: Healthcare (Basel). 2023 Sep 28;11(19):2645. doi: 10.3390/healthcare11192645 (PMC10572283; doi:10.3390/healthcare11192645)
Supplement: Supplementary file 1 [file healthcare-11-02645-s001.zip › Supplementary file S2. Methodological rigour JCM.pdf]

File S2. Strategies for methodological rigour.

Credibility[42]
• Triangulation of information sources: registered nurses and nursing assistants. Allows to investigate different perspective of the phenomenon.<br>• Triangulation of the interview analysis process: two independent coders for each interview. Allows to improve the internal validity of code assignment to the discourses.<br>• Interviewees' feedback: in the cases where contact data were available, the analysed interviews were sent there to get feedback of possible transcription and/or interpretation mistakes.<br>• Implementation of saturation criterion during the analysis process.<br>• Protection of the interviewees' anonymity and confidentiality.
Transferability[36]
• Purposive sampling of maximum variation. Allows to include the common spectrum of professional profiles, selecting interviewees able to describe the studied phenomenon. Furthermore, maximum variation of the professionals' characteristics (see Table 1) allows to record maximum diversity of the phenomenon.<br>• Completeness of collected data and their abundance and diversity, allowing a detailed description of the phenomenon.<br>• Variability of the features of the studied units. Allows to consider contextual differences in data interpretation.
Consistence[42,45]
• Detailed description of interviewees, data collection methods and analysis process.<br>• Traceability documents available for auditory on the part of external reviewers (in Spanish).
Confirmability: neutrality of investigators and bracketing[45].

- Bracketing process. A suspension of beliefs, prejudices and previous values about care of awake and intubated patients was performed before and after the interviews, before and after the analysis process and during the composition of the results. An external observer compared previous description of investigators' own experience in care of those patients -in the form of accounts and/or field journals- with the progressively obtained findings until the end of the study; this allowed to rule out prejudices influence on the results and keep optimal control of the phenomenological reduction conditions.
- Description of the researcher's relationship (main researcher) with the interviewees: absence of a previous relationship of the interviewer with the interviewees avoided an influence of personal bias or preconceptions in the interview process.
