# Supplementary material for: Determinants of Communication Failure in Intubated Critically Ill Patients: A Qualitative Phenomenological Study from the Perspective of Critical Care Nurses
Source: Healthcare (Basel). 2023 Sep 28;11(19):2645. doi: 10.3390/healthcare11192645 (PMC10572283; doi:10.3390/healthcare11192645)
Supplement: Supplementary file 1 [file healthcare-11-02645-s001.zip › Supplementary File S3 JCM.pdf]

Supplementary file S3. COREQ Checklist

Consolidated criteria for reporting qualitative studies (COREQ): 32-item checklist

Developed from:
Tong A, Sainsbury P, Craig J. Consolidated criteria for reporting qualitative research (COREQ): a 32-item checklist for interviews and focus groups. *International Journal for Quality in Health Care*. 2007. Volume 19, Number 6: pp. 349 – 357

YOU MUST PROVIDE A RESPONSE FOR ALL ITEMS. ENTER N/A IF NOT APPLICABLE

No. Item	Guide questions/description	Reported on Page #
**Domain 1: Research team and reflexivity**		
Personal Characteristics		
1. Inter viewer/facilitator	Which author/s conducted the inter view or focus group?	#6 (2.Method, 2.4 Data collection)
2. Credentials	What were the researcher's credentials? E.g. PhD, MD	#6 (2.Method, 2.4 Data collection)
3. Occupation	What was their occupation at the time of the study?	#6 (2.Method, 2.4 Data collection)
4. Gender	Was the researcher male or female?	#6 (2.Method, 2.4 Data collection)
5. Experience and training	What experience or training did the researcher have?	#6 (2.Method, 2.4 Data collection)
Relationship with participants		
6. Relationship established	Was a relationship established prior to study commencement?	#7 (2.Method, 2.4 Data collection)
7. Participant knowledge of the interviewer	What did the participants know about the researcher? e.g. personal goals, reasons for doing the research	#6 (2.Method, 2.4 Data collection)
8. Interviewer characteristics	What characteristics were reported about the inter viewer/facilitator? e.g. Bias, assumptions, reasons and interests in the research topic	#6 (2.Method, 2.4 Data collection)
**Domain 2: study design**		
Theoretical framework		
9. Methodological orientation and Theory	What methodological orientation was stated to underpin the study? e.g. grounded theory, discourse analysis, ethnography, phenomenology, content analysis	#5 (2.Method, 2.1 Design)
Participant selection		

10. Sampling	How were participants selected? e.g. purposive, convenience, consecutive, snowball	#5 (2.Method, 2.2 Participants recruitment)
11. Method of approach	How were participants approached? e.g. face-to-face, telephone, mail, email	#6 (2.Method, 2.4 Data Collection)
12. Sample size	How many participants were in the study?	#9 (3. Findings)<br>#29 (Table 1)
13. Non-participation	How many people refused to participate or dropped out? Reasons?	#7 (2.Method, 2.5 Ethical considerations)
Setting		
14. Setting of data collection	Where was the data collected? e.g. home, clinic, workplace	#6-7 (2.Method, 2.3 Settings and 2.4 Data Collection)
15. Presence of non-participants	Was anyone else present besides the participants and researchers?	#7 (2.Method, 2.4 Data collection)
16. Description of sample	What are the important characteristics of the sample? e.g. demographic data, date	#9 (3. Findings)<br>#29 (Table 1)
Data collection		
17. Interview guide	Were questions, prompts, guides provided by the authors? Was it pilot tested?	Supplementary file 1<br>#6 (2.Method, 2.4 Data collection)
18. Repeat interviews	Were repeat inter views carried out? If yes, how many?	N/A
19. Audio/visual recording	Did the research use audio or visual recording to collect the data?	#7 (2.Method, 2.4 Data collection)
20. Field notes	Were field notes made during and/or after the inter view or focus group?	#7 (2.Method, 2.4 Data collection)
21. Duration	What was the duration of the inter views or focus group?	#7 (2.Method, 2.4 Data collection)
22. Data saturation	Was data saturation discussed?	#9 (2.<br>Method, 2.7 Trusthworthiness)<br>Supplementary file 2
23. Transcripts returned	Were transcripts returned to participants for comment and/or correction?	#9 (2. Method,<br>2.7 Trusthworthiness) and Supplementary file 2
**Domain 3: analysis and findings**		
Data analysis		
24. Number of data coders	How many data coders coded the data?	#8 (2. Method, 2.6 Data Analysis<br>#29 (Table 1)
25. Description of the coding tree	Did authors provide a description of the coding tree?	#9 and Figure 1<br># 11 and Figure 2<br># 14 and Figure 3
26. Derivation of themes	Were themes identified in advance or derived from the data?	#8 (2. Method, 2.6 Data Analysis)

27. Software	What software, if applicable, was used to manage the data?	#8 (2. Method, 2.6 Data Analysis)
28. Participant checking	Did participants provide feedback on the findings?	Supplementary file 2
Reporting		
29. Quotations presented	Were participant quotations presented to illustrate the themes/findings? Was each quotation identified? e.g. participant number	Tables 2, 3, 4. (3. Findings)
30. Data and findings consistent	Was there consistency between the data presented and the findings?	# 9-18 and #18-23 (3. Findings and 4. Discussion)
31. Clarity of major themes	Were major themes clearly presented in the findings?	# 9-18 (3. Findings)
32. Clarity of minor themes	Is there a description of diverse cases or discussion of minor themes?	# 9-18 (3. Findings and 4. Discussion)
